# Do the ends justify the means? Impact of drought progression rate on stress response and recovery in *Vitis vinifera*

**DOI:** 10.1111/ppl.13590

**Published:** 2021-11-10

**Authors:** Cristina Morabito, Jessica Orozco, Giulia Tonel, Silvia Cavalletto, Giovanna Roberta Meloni, Andrea Schubert, Maria Lodovica Gullino, Maciej Andrzej Zwieniecki, Francesca Secchi

**Affiliations:** ^1^ Department of Agriculture, Forest and Food Sciences University of Turin Grugliasco Italy; ^2^ Department of Plant Sciences University of California Davis Davis California USA; ^3^ Agroinnova, Centre of Competence for Innovation in the Agro‐Environmental Field Grugliasco Italy

## Abstract

Plants are frequently exposed to prolonged and intense drought events. To survive, species must implement strategies to overcome progressive drought while maintaining sufficient resources to sustain the recovery of functions. Our objective was to understand how stress rate development modulates energy reserves and affects the recovery process. Grenache *Vitis vinifera* cultivar was exposed to either fast‐developing drought (within few days; FDD), typical of pot experiments, or slow‐developing drought (few weeks, SDD), more typical for natural conditions. FDD was characterized by fast (2–3 days) stomatal closure in response to increased stress level, high abscisic acid (ABA) accumulation in xylem sap (>400 μg L^−1^) without the substantial changes associated with stem priming for recovery (no accumulation of sugar or drop in xylem sap pH). In contrast, SDD was characterized by gradual stomatal closure, low ABA accumulation (<100 μg L^−1^) and changes that primed the stem for recovery (xylem sap acidification from 6 to 5.5 pH and sugar accumulation from 1 to 3 g L^−1^). Despite FDD and SDD demonstrating similar trends over time in the recovery of stomatal conductance, they differed in their sensitivity to xylem ABA. Grenache showed near‐isohydric and near‐anisohydric behavior depending on the rate of drought progression, gauging the risk between hydraulic integrity and photosynthetic gain. The isohydry observed during FDD could potentially provide protection from large sudden swings in tension, while transitioning to anisohydry during SDD could prioritize the maintenance of photosynthetic activity over hydraulic security.

## 1 INTRODUCTION

Over the course of their life, plants experience a wide range of climatic conditions, fluctuations in temperature, nutrient, and water availability that are often suboptimal and can severely constrain their growth and reproductive development (Yuan et al. 2019; Zeppel et al. 2014). In particular, perennial species are left increasingly vulnerable to additional abiotic and biotic stressors, greatly limiting productivity (Allen et al. 2010). Among abiotic stressors, drought is the most pervasive; plants recurrently face alternating periods of drought, varying in length and intensity, followed by the sudden availability of water, often in the form of rain. Typically, under natural conditions, slow‐developing drought spans weeks, if not months (Zargar et al. 2011). Initially, the onset of drought leads to a drop in plant water potential and stomatal conductance, consequently hindering photosynthesis and impeding growth. Prolonged exposure to stress results in xylem embolism formation, thus interrupting and/or completely halting water transport, which, if not ameliorated, may culminate in plant death (Tyree & Sperry 1989; Zwieniecki & Secchi 2015). Plant stress response strategies emerged to account for drought severity, duration, and frequency typical of their respective environments, with the goal of utilizing the sudden burst of water supply to resume physiological activity. Therefore, survival strategies to cope with drought stress cannot just be seen as passive but rather as proactive preparations for recovery prompted by the sudden availability of water. This novel premise stipulates that drought and recovery are not dichotomous and independent but should be considered as one interwoven continuous process (Ruehr et al. 2019).

Processes that lay the foundation for facilitating recovery may be activated alongside conventional stress response mechanisms. Given that preparation for recovery is initiated during drought, its course, and effectiveness may be impacted by features characteristic of temporal stress dynamics such as rate and duration, which may dictate the ultimate success of post‐drought recovery (Anderegg et al. 2013). Much of our current understanding rests upon studies performed on plants often maintained in pots and greenhouses, where drought is simulated by an abrupt discontinuity in water supply, which can skew or even completely overlook the processes at play during the natural trajectory of drought stress (Romero et al. 2017). In nature, stress usually develops gradually over weeks or months as the effective soil volume per plant is large, while large negative tensions are often achieved in a matter of days or even hours in experimental settings, thus altering or inhibiting acclimation responses and limiting our ability to reliably assess recovery dynamics (Ingrisch & Bahn 2018). In order to survive, species must be able to coordinate an arsenal of multiscale responses, including adjustments to their biochemistry and physiology that can concurrently address the progressive drought while maintaining sufficient resources to sustain the prospective recovery of plant function. These adjustments include changes in the level of stress hormones (Daszkowska‐Golec & Szarejko 2013), osmolytes and protective chemicals (Blum 2017), xylem sap pH (Secchi & Zwieniecki 2012), metabolism of nonstructural carbohydrates (NSC) (Tomasella et al. 2019; Trifilo et al. 2017), and expression of genes (Cramer et al. 2007). Therefore, the length and severity of stress incurred by plants can have downstream ramifications on the degree and path of recovery. How and which aspects of stem biochemistry and whole plant physiology are affected by the rate of drought stress progression and how these changes impact recovery remains an open question.

Mounting evidence points to the linkage between NSC metabolism and a plant's capacity to cope and recover from drought stress (O'Brien et al. 2014; Pratt et al. 2021; Schwalm et al. 2017; Trugman et al. 2018). Amidst periods of water scarcity, during which stomatal closure prevents photosynthetic carbon uptake (McDowell et al. 2008, 2011), stored NSC can act as a buffer providing carbon to maintain basic metabolism and defense processes (McDowell & Sevanto 2010; Sala et al. 2012). Drought affects not only the total carbohydrates amount but also the allocation and composition of NSCs, all of which can be linked to concurrent changes in xylem sap chemistry (Ivanov et al. 2019; Savi et al. 2016; Tomasella et al. 2017). Specifically, in some species, a drop in xylem sap pH induces an accumulation of soluble sugars in the apoplast that can promote recovery by serving as osmolytes generating a gradient to refill embolized conduits; processes that are associated with “stem priming” for recovery (Pagliarani et al. 2019; Secchi & Zwieniecki 2012; Tomasella et al. 2021). Given that sugar depletion is expected during prolonged drought stress, rapid recovery of plant photosynthetic capacity might be a crucial adaptation that would confer a competitive advantage to plants. However, a gradual reinstatement of pre‐stress functions may be necessary to afford sufficient time to repair drought stress‐related damages. For example, delaying stomatal opening despite tension alleviation may reduce the transpirational demand and provide additional time for the slow osmotically driven removal of embolism to occur. In this respect, ABA‐mediated control of stomatal aperture may be more important over passive turgor and water potential‐driven responses. Thus, the objective of this study was to understand how plants use their time under stress to modulate their energy reserves (mostly carbohydrate supply) and xylem chemistry (pH and ABA content) to enhance recovery processes.

As sessile organisms subjected to a wide range of constantly changing environmental conditions, plants' survival depends on their capacity to integrate information about their surroundings, gauge potential tradeoffs and adjust their physiology accordingly while optimizing resources, especially under water stress. Therefore, mechanisms that regulate gas exchange and restrict water loss are imperative for adapting and thriving in these conditions. While duration and magnitude are defining features of drought, the rate of stress progression is seldom considered despite its potential importance for the acclimation of plants to stress and their subsequent recovery. Therefore, we hypothesized that a slow‐developing drought (SDD) would allow the xylem sap chemistry to change in time, hence priming the stem for recovery while delaying stomatal opening. A fast‐developing drought (FDD) would only prioritize the conservation of water by shutting stomata without the changes associated with stem priming. Given that stomatal behavior can have large implications for NSC and water‐loss dynamics, we tested our hypothesis on *Vitis vinifera* cultivar Grenache purported to be near‐isohydric (Shelden et al. 2017). We evaluated stress responses and recovery by analyzing both physiological and chemical parameters in response to fast and natural timing of drought occurrence, thus investigating the specific drought response strategies.

## 2 MATERIALS AND METHODS

### 2.1 Plant materials and experimental set up

*Vitis vinifera* cv Grenache cuttings were provided by the nursery Vivai Cooperativi Rauscedo‐San Giorgio della Richinvelda (PN), Italy. These commercially available plants are typically grafted on rootstock 1130P (*V. berlandieri* cv. Resseguier nr. 2 × *V. rupestris* cv. Du Lot.). In this study, we refer to the plants simply as Grenache.

Two‐year‐old grapevines were grown in a greenhouse under partially controlled climatic conditions. Temperature and relative humidity were maintained during the experiment in the range of 22°C–31°C and 40%–80%, respectively (daily average temperature and relative humidity are reported in Table S1). Natural daylight was supplemented when necessary with light from metal halogen lamps, maintaining a minimum of 500–600 mmol photons m^−2^ s^−1^ during a 12‐h‐light/12‐h‐dark cycle. Each plant grew in a 4‐L pot filled with a substrate composed of sandy‐loam soil/expanded clay/peat mixture (2:1:1 by weight). A total of 25 grafted grapevines with 2–5 branches per plant were used in this study. At the beginning of the experiment, Grenache plants had an average length of 87.05 ± 11.8 cm (as measured from the graft union) and were characterized by the same phenological phase, with at least 10 fully expanded leaves at the scion assuring its photosynthetic independence. Furthermore, the plants remained in a vegetative phenological state, typical of 2‐year‐old plants, through the extent of the experiment.

The 25 grapevines were further divided into three groups: 10 plants were exposed to SDD treatment, 10 plants to a FDD, and the remaining 5 grapevines were kept as controls plants (CTR) irrigated to field capacity every morning during the whole experimental period. The SDD was achieved by progressively reducing the amount of water provided to the plants (20% less water used every day), while the FDD was induced by interrupting irrigation (Figure S1). The daily water loss for both FDD and SDD is shown in Figure S2. In both treatments, water stress was imposed until stem water potential reached an average level of −2 MPa. Once water stress levels were reached, the grapevines were re‐watered in the morning up to field capacity and for the following 10 days (REC).

Xylem sap and stem tissues were collected from the treated (SDD, FDD, and REC) and control plants (CTR) throughout the experiment duration and the samples were stored for further chemical analyses. Physiological parameters (stem water potential, stomatal conductance, and photosynthesis) were monitored during the entire experiment (i.e. from the start of the stress treatments until full recovery of physiological functions) in both drought and control plants. Since sampling for the biochemical properties of xylem sap was destructive, we randomly removed at least three lateral branches per treatment (one branch per plant) on every sampling date. Sample pooling was every 6 days for FDD, encompassing three periods (FD1–FD3), and every 12 days for SDD, encompassing four periods (SD1–SD4). During recovery, since stem water potential recovered within 1 day for both treatments, samples were collected and grouped within the first day (FR1, SR1) and a second sample aggregation was done for the remaining recovery period (FR2, SR2).

### 2.2 Measurement of leaf gas exchange and xylem pressure

Stomatal conductance (*g*_*s*_) and net photosynthesis (*A*_*n*_) were measured on fully expanded leaves exposed to direct sunlight, using a portable infrared gas analyzer (ADC‐LCPro+ system, The Analytical Development Company Ltd). Measurements were performed using a 6.25 cm^2^ leaf chamber equipped with artificial irradiation (1200 μmol photon m^−2^ s^−1^), set with a chamber temperature of 25°C to avoid overheating. CO_2_ values were maintained at greenhouse conditions (400–450 ppm). Leaf gas exchange was monitored daily (between 10:00 and 12:00 h) on three to five plants in each treatment (one leaf per plant) for the whole duration of the experimental trial. Meanwhile, three leaves per treatment (each from different plants) were collected every 2–3 days for xylem pressure measurements (stem water potential).

Xylem pressure measurements were performed on fully expanded non‐transpiring leaves. Prior to taking the measurements, leaves were placed in humidified aluminum foil‐wrapped plastic bags for 20 min before excision. After excision, leaves were allowed to equilibrate for an additional 15 min and water potential was measured using a Scholander‐type pressure chamber (Soil Moisture Equipment Corp.).

### 2.3 Sap and stem sampling procedure

Xylem sap was collected from treated (SDD, FDD, and REC) and control (CTR) plants, according to a previously described method (Secchi & Zwieniecki 2012). Briefly, a branch was attached through a plastic tube to a syringe needle. The needle was threaded through a rubber cork to a vacuum chamber, with the needle tip placed in a 1.5‐mL plastic tube. After a vacuum suction was generated, pieces of stem were consecutively cut from the top, allowing liquid from open vessels to be sucked out of the stem and collected in the tube. Sap samples were stored at −20°C until analyses of pH and NSC content were conducted.

The stems sampled for sap collection were cut in small sections using a fresh razor blade and microwaved at 700 W for 3 min to stop enzymatic activities. Samples were then oven‐dried at 70°C for 24 h, ground to fine powder (particle size <0.15 mm) using a tissue lyser system (TissueLyser II, Qiagen), and kept for further analyses (starch and soluble sugar content) at room temperature.

### 2.4 Measurements of pH and soluble carbohydrates in xylem sap

Variations in xylem sap pH during SDD and FDD and along recovery period compared with control plants were evaluated using a micro pH electrode (PerpHect ROSS, Thermo Fischer Scientific). Non‐structural carbohydrates (NSC) content in xylem sap samples was quantified following the anthrone‐sulfuric acid assay described by Leyva et al. (2008) with the modifications indicated in Secchi and Zwieniecki (2012). In short, 50 μL samples were mixed with 150 μL of anthrone in sulfuric acid (0.1%, w/v) in a 96‐well micro‐plate (iMark Microplate Absorbance Reader, BioRad). The plate was cooled on ice for 10 min, heated at 100 °C for 10 min, and then equilibrated to room temperature for 10 min. A glucose standard curve was used to compare the colorimetric response of the samples, whose absorbance was read at 620 nm. Soluble carbohydrates concentration was expressed as g L^−1^ of glucose.

### 2.5 Analysis of soluble sugars and starch concentration in stem samples

Powdered sample materials (25 ± 4 mg) were transferred into a 1.5 mL Eppendorf test tube. To extract soluble sugars, 1 mL of 0.2 M sodium acetate buffer solution (pH = 5.5) was added to each sample, vortexed and incubated at 70°C for 10 min. The NSC were quantified following the procedure described above and the sugar concentration was expressed as mg g^−1^ dry weight. For starch analyses, the remaining pellet was exposed to 100°C for 10 min and submitted to enzymatic digestion for 4 h at 37°C in 0.2 M sodium acetate buffer (pH = 5.5) with 0.7 U of amylase and 7 U of amyloglucosidase. Once the digestion was completed, samples were centrifuged for 5 min at 21,000*g*, and the supernatant was diluted 1:20 and quantified using the method described above for determining soluble carbohydrates content.

### 2.6 HPLC‐MS/MS analysis of sap ABA content

ABA concentration was quantified following the method described by Siciliano et al. (2015) with minor changes. Xylem sap samples were centrifuged at 13,000*g* for 5 min at 4°C. From the obtained supernatant, a total volume of 50 μL for each sample was collected in a 1 mL amber glass vial containing an appropriate glass insert (Supelco, Sigma‐Aldrich) for small sample volumes and analyzed by HPLC‐MS/MS. High‐Performance Liquid Chromatography was carried out using a 1260 Agilent Technologies system equipped with a binary pump and a vacuum degasser. Sample aliquots (20 μL) were injected on a Luna C18 (150 × 2 mm i.d., 3 μm Phenomenex) and ABA was eluted in isocratic conditions of 65:35 (H_2_O:CH_3_CN v/v acidified with HCOOH 0.1%) under a flow of 200 μL min^−1^ for 5 min. Using an electrospray (ESI) ion source operating in negative ion mode, samples were introduced into a triple‐quadruple mass spectrometer (Varian 310‐MS TQ Mass Spectrometer). Analyses were conducted in MRM mode using two transitions: 263 > 153 (CE 12 V) for quantification, 263 > 219 (CE 12 V) for monitoring, with 2 mbar of Argon (Ar) as collision gas. The external standard method was applied to quantify ABA concentration in target samples. In detail, a standard curve was generated using an original ABA standard (Sigma Aldrich; purity 98.5%), with concentrations ranging from 10 to 500 μg L^−1^. The detection (LOD) and quantification (LOQ) limits were calculated based on the standard deviation of the response (*σ*) and slope of the calibration curve (*S*) ratio in accordance with the ICH Harmonized Tripartite Guideline expressed as: LOD = 3.3*σ*/*S*; LOQ = 10*σ*/*S*. Calculated final values were as follows: LOD = 0.87 ng mL^−1^; LOQ = 2.90 ng mL^−1^.

### 2.7 Statistical analyses

Significant differences among treatments were analyzed by applying a one‐way analysis of variance. Fisher LSD significant difference post‐hoc test was used for separating means when analysis of variance results was significant (*P* < 0.05). The SPSS statistical software package (v24.0, SPSS Inc.) was used to run the statistical analyses, and Sigma Plot software (Systat Software Inc.) was used to create figures.

## 3 RESULTS

### 3.1 Physiological changes in response to SDD, FDD, and recovery

**FIGURE 1:**
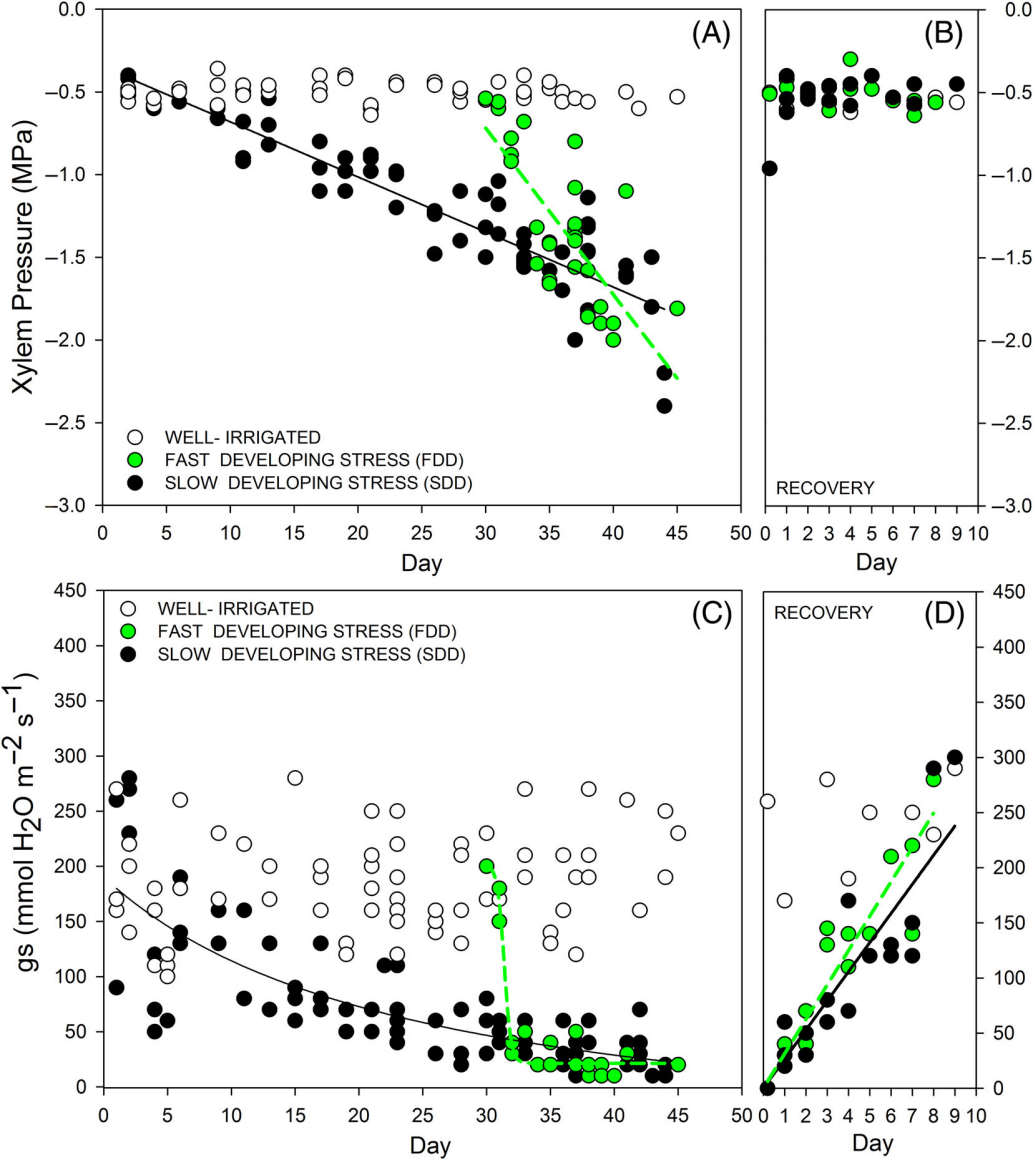
Temporal dynamics of stem water potential (xylem pressure; A,B) and stomatal conductance (*g*_*s*_; C,D) during fast‐developing drought (FDD) and slow‐developing drought (SDD), and during stress recovery. Each circle represents a plant

**FIGURE 2:**
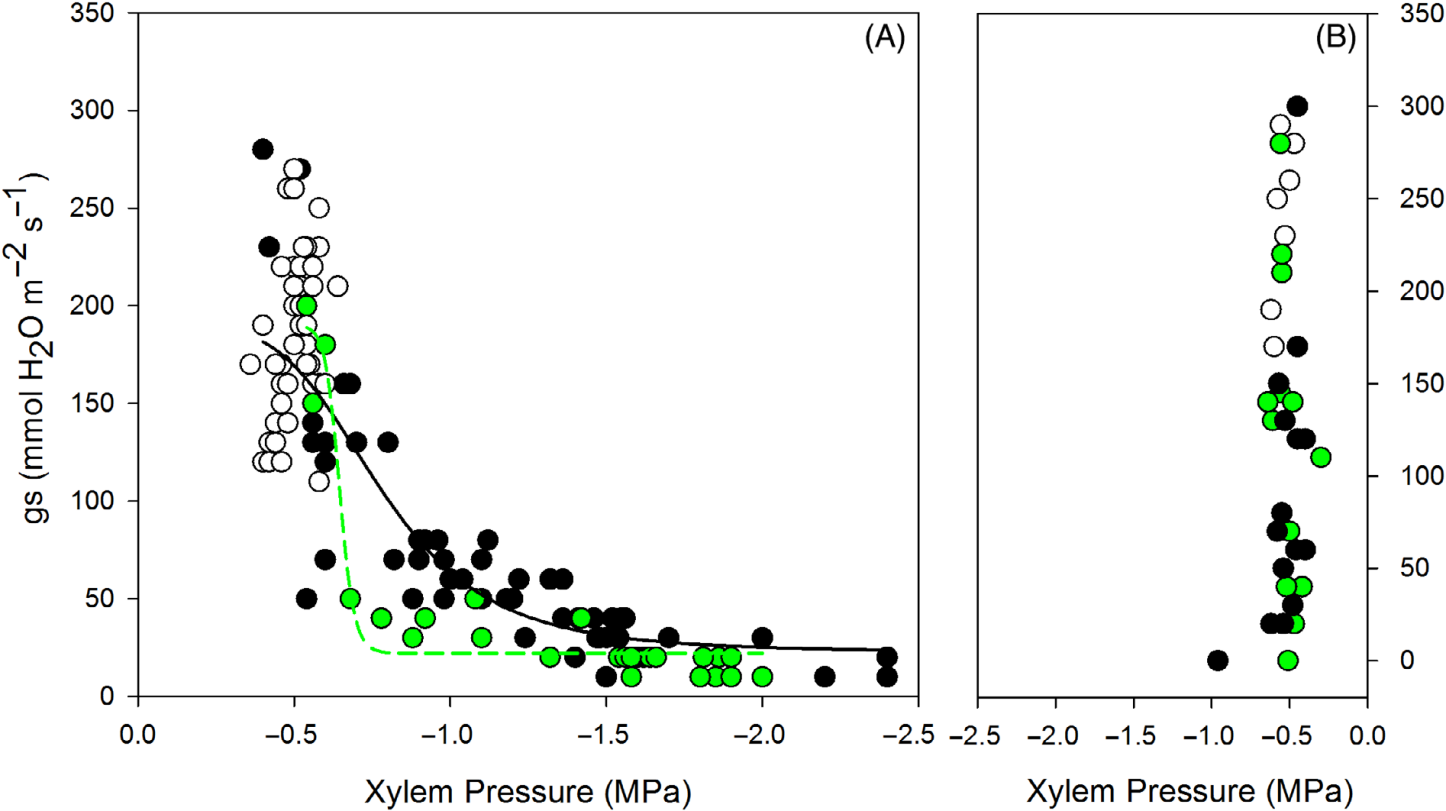
Stomatal conductance (*g*_*s*_) in relation to xylem pressure in grenache plants during (A) fast‐developing drought (FDD) and slow developing drought (SDD) and during (B) recovery from FDD and SDD. Data were fitted with the four‐parameter logistic curves (dose–response curve; black and green lines for, respectively, SDD and FDD treatment; for more details see Secchi & Zwieniecki 2014). Parameters that describe curves for the two stressed populations are statistically different (FDD‐EC50*g*_*s*_ = −0.645 MPa and SDD‐EC50*g*_*s*_ = −0.777 MPa; Paternoster *t* test, *P* < 0.005)

Using two distinct methods to impose drought onto potted plants: (1) an immediate interruption of irrigation resulting in fast‐developing drought (FDD) and (2) a constant reduction in available water resulting in slow‐developing drought (SDD), we successfully implemented two rates of drought progression allowing us to test the proposed hypotheses. Grenache plants exposed to SDD reached the stress level of approximately −2 MPa in 44 days (ψ stem: −2.06 ± 0.40 MPa), while water stress was achieved within 18 days in FDD (ψ stem: −1.85 ± 0.07 MPa; Figure 1A). The rate of water stress progression was significantly different between treatments and was ~0.09 and ~0.025 (MPa day^−1^), respectively, for FDD and SDD (Figure 1A). Stomatal conductance (*g*_*s*_) progressively decreased during SDD treatment, while it seemed to collapse within 1 day in FDD treatment (Figure 1C). The response of *g*_*s*_ to xylem pressure was different between the two treatments; in the SDD treatment, plants gradually shut stomata in response to increment of stress level and remain partially open even at −1 MPa, while stomata closure occurred at the onset of low stress around −0.6 MPa in FDD (Figure 2A). Post‐rewatering, water potential recovered within the first day in both treatments (Figure 1B). Recovery of *g*_*s*_ was much slower than that of water potential but was not significantly affected by treatment (Figure 1D). Recovery of *g*_*s*_ expressed as a response to water potential revealed no relationship (Figure 2B).

As expected, net photosynthesis and stomatal conductance were well correlated; a constant reduction of net photosynthesis was in fact coupled with a progressive reduction of *g*_*s*_ (Figure S3A). Rewatering completely restored photosynthetic activity to pre‐stress measurements after stomata were fully open (Figure S3B).

**FIGURE 3:**
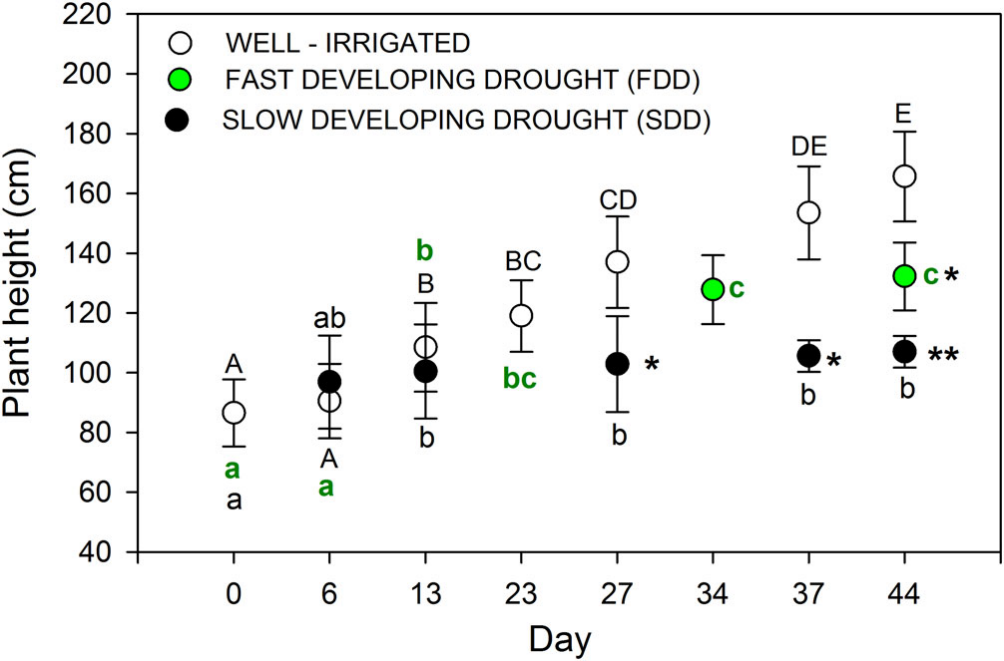
Temporal plant growth during fast‐developing drought (FDD; green circles) and slow‐developing drought (SDD; black circles). White circles denote well‐irrigated grapevine plants (CTR). One‐way ANOVA tests suggest significant differences among the periods of experiment duration (days) and among the three treatments (*P* < 0.05). Among time, letters denote homogeneous groups based on the Fisher LSD method (uppercase letters, differences among well‐irrigated plants; lowercase letters, differences among SDD plants and green letters, differences among FDD plants). Asterisks denote significant differences among treatments on the same date. Data are mean values and bars are SE (control: *N* = 5 plants; SDD and FDD: *N* = 10 plants)

At the end of the treatments, well‐irrigated plants were longer than the stressed ones (165.67 ± 15.04 cm versus 107 ± 5.3 cm and 132.25 ± 11.32 cm, respectively, for SDD and FDD treatments). The plants exposed to SDD at the end of the experiment showed a 23% increment in stem length, while Grenache exposed to FDD were about 52% longer than plants at the beginning of the experiment (Figure 3). Moreover, SDD grapevines grew only the first 13 days from the beginning of the stress.

### 3.2 Biochemical changes in xylem sap in response to stresses and recovery

**FIGURE 4:**
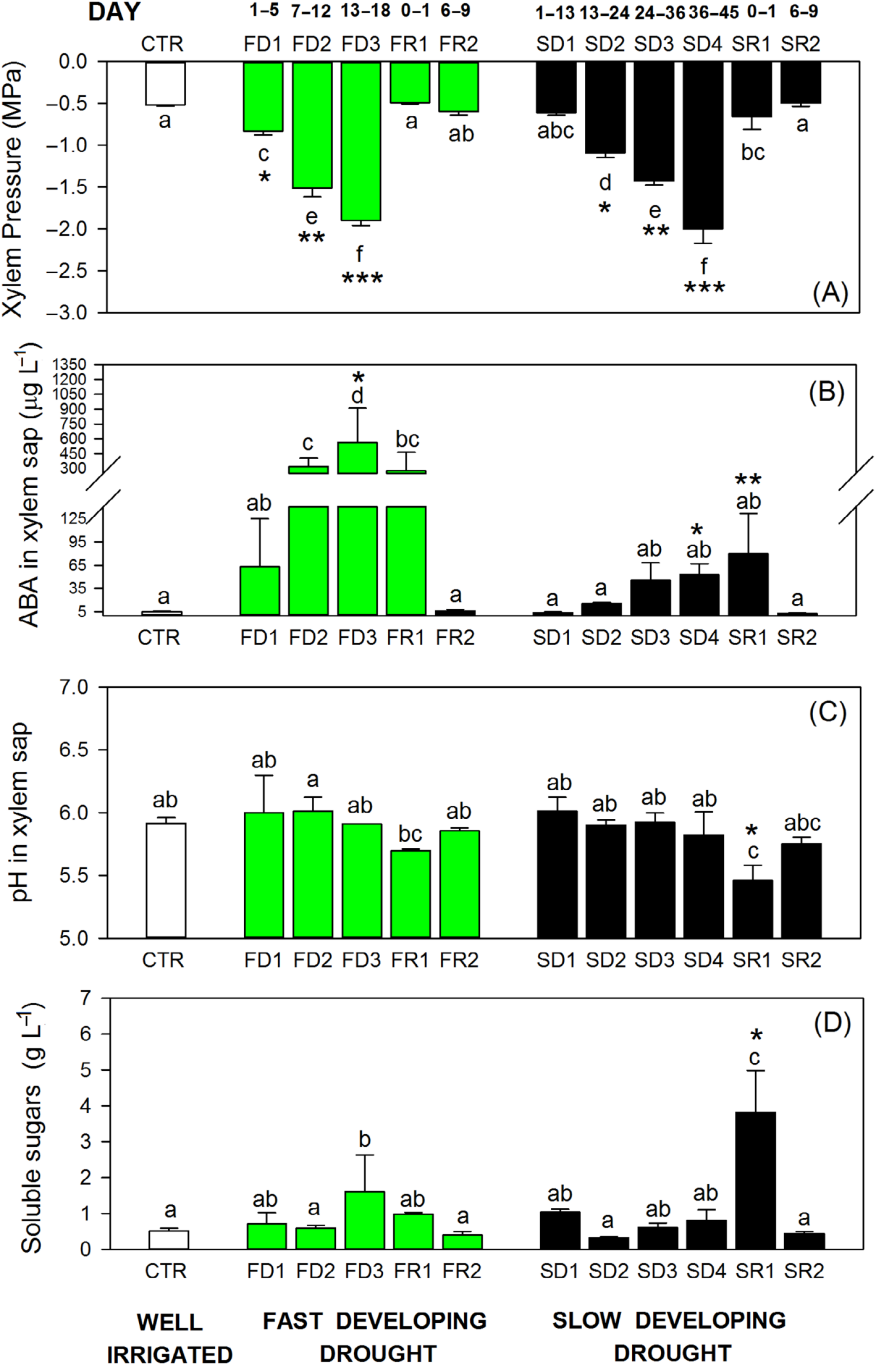
(A) Xylem pressure, (B) abscisic acid (ABA) concentration, (C) pH values, and (D) soluble sugar content measured from xylem sap collected from plants exposed to a fast‐developing drought (FDD; green bars) and to a slow‐developing drought (SDD; black bars). White bars indicate average values measured in well‐irrigated plants (CTR). One‐way ANOVA test suggests significant differences during the imposition of the stresses (*P* < 0.05). Letters denote homogeneous groups based on the Fisher LSD method. Asterisks denote significant differences in SDD and in FDD groups; data are mean values and bars are SE (*n* = 3 replicates with a pool of minimum two plants each)

The ABA in xylem sap increased with the increment of drought level for both stresses (Figure 4B; Figure S3B), but the highest ABA accumulation was measured at the end of FDD treatment (FD3, Figure 4B). The ABA accumulation to 60–90 μg L^−1^ forced complete stomatal closure (Figure 1C), but ABA continued to accumulate under FDD treatment and reached values 10 times higher than those under SDD conditions. During the recovery phase, xylem pressure recovered within 1 day, while ABA concentrations during the few hours and 1 day post‐recovery (R1, Figure 4B) remained high at the level of FDD and SDD under drought. The drop in ABA content did not occur until 8–10 days post rehydration. At that time, ABA concentration decreased to pre‐stress values in both treatments (R2, Figures 4B; Figure S4B).

**FIGURE 5:**
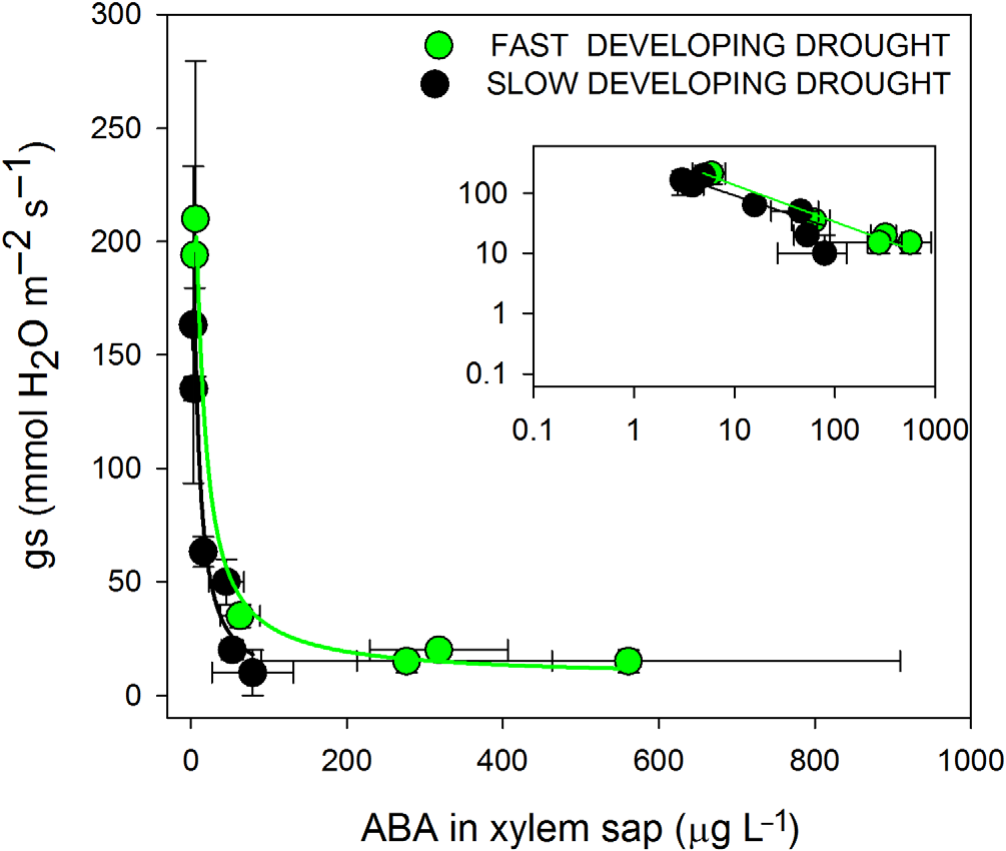
Relationship between stomatal conductance (*g*_*s*_) and abscisic acid (ABA) content in xylem sap during fast‐developing drought (FDD, green circles) and slow‐developing drought (SDD; black circles). Insets depict stomatal conductance (*g*_*s*_) versus ABA represented with log scale values. Green lines represent the curves obtained for FDD and black lines for SDD treatment. Data are mean values and bars are SE (*n* = 3 replicates with a pool of minimum two plants each)

During FDD and SDD, the response of ABA to stomatal conductance was well correlated in both drought treatments; FDD: *R* ^2^ = 0.98; *P* < 0.001, SDD: *R* ^2^ = 0.92; *P* < 0.001 (Figure 5). The ABA concentration increased significantly in the xylem sap of the stems while *g*_*s*_ decreased and a maximum level of ABA was reached when stomata were closed.

**FIGURE 6:**
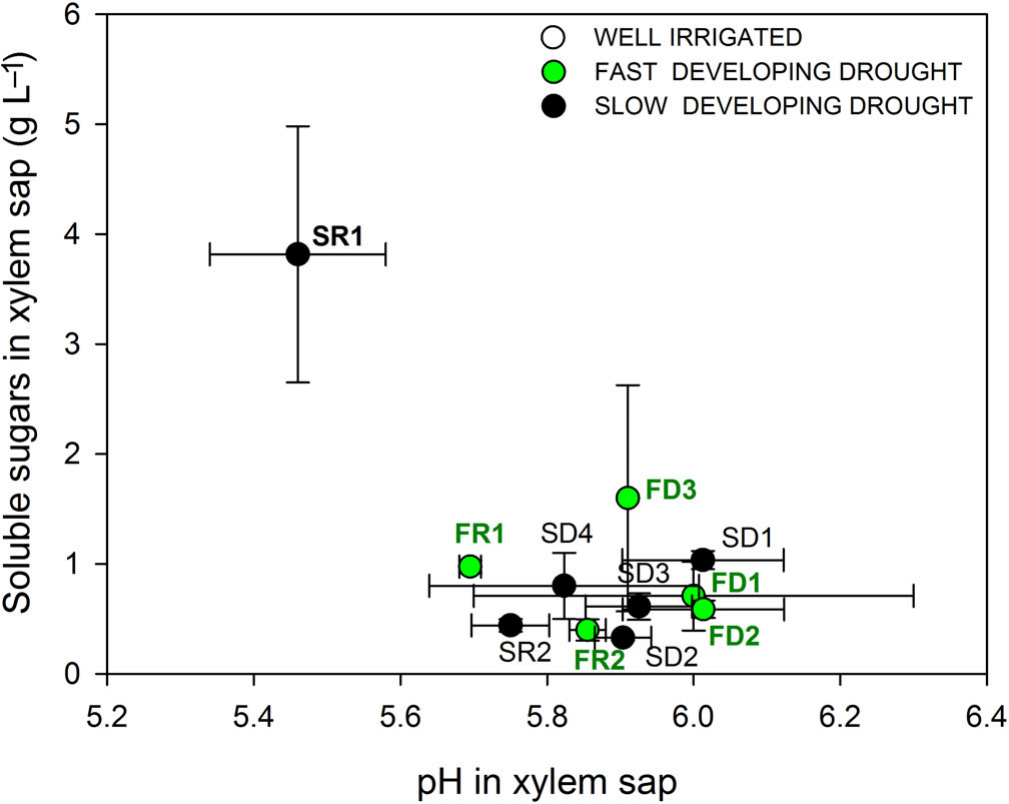
Xylem soluble sugar content related to pH values during fast‐developing drought (FDD; green circles) and slow‐developing drought (SDD; black circles). Data are mean values and bars are SE (*n* = 3 replicates with a pool of minimum to plants each)

Fast‐developing drought did not significantly affect the pH of xylem sap. Sap acidification was observed after 1 day of relief from slow‐developing drought (Figure S4). Xylem sap pH changed from 5.92 ± 0.042 in control plants to 5.46 ± 0.12 in recovered plants (Figure 4C). The acidification of xylem sap occurred in parallel with a significant increase in soluble carbohydrate content (from 0.51 ± 0.079 g L^−1^ in control plants to 3.82 ± 1.16 g L^−1^ in recovered plants; Figure 4D). The total amount of carbohydrates in the sap returned to pre‐stress levels after 10 days of rehydration (0.44 ± 0.056 g L^−1^), when pH values were higher and overlapping those of irrigated plants (pH: 5.75 ± 0.053; Figure 4D). During the drought experiments, sugar concentration was low and not correlated with pH values (Figure 6). However, Grenache SDD‐stressed plants showed high carbohydrate content only at lower pH values during the first day of recovery (SR1), which was significantly different from the rest of the measurements (Figure 6).

### 3.3 Biochemical changes in stem tissues in response to stresses and recovery

**FIGURE 7:**
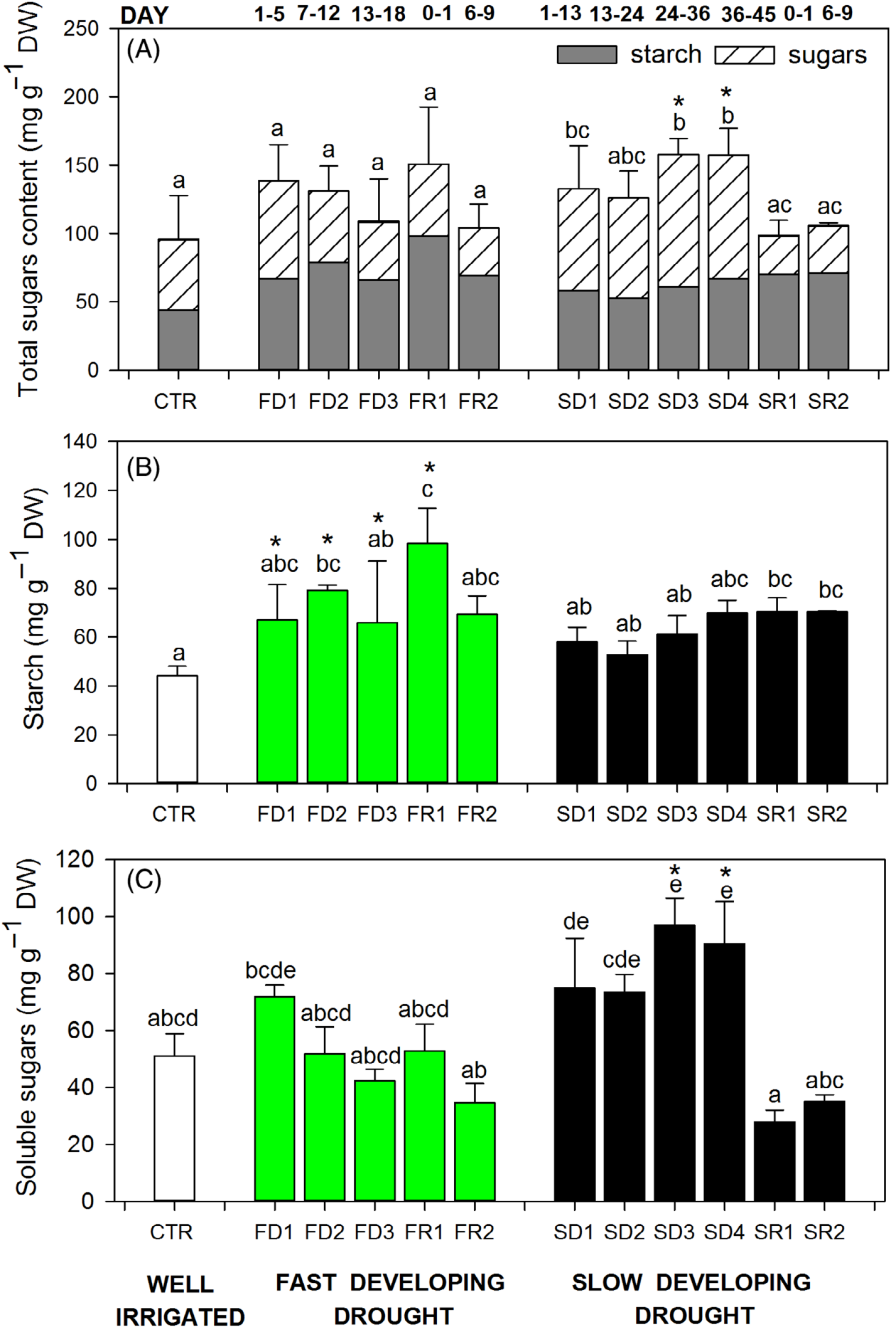
(A) Total carbohydrates (starch plus soluble sugars), (B) starch content, and (C) soluble sugars content measured from stem tissues collected from plants exposed either to a fast‐developing drought (FDD; green bars) or to a slow‐developing drought (SDD; black bars). White bars indicate average values measured in well‐irrigated plants (CTR). One‐way ANOVA test suggests significant differences during the imposition of the stresses (*P* < 0.05). Letters denote homogeneous groups based on the Fisher LSD method. Asterisks denote significant differences in SDD and in FDD groups; data are mean values and bars are SE (*n* = 3 replicates with a pool of minimum two plants each)

Drought treatments affected differently the total NSC contents in the stems. Plants exposed to FDD treatment did not modify the total carbohydrates (starch plus soluble sugars) content in stem tissues during both stress and recovery (Figures 7A; Figure S5). However, plants exposed to SDD showed an increase of total NCS content during stress, and the values returned to pre‐stress levels when water was alleviated, with sugar concentration overlapping those of well‐watered grapevines (SDD: 94.32 ± 26.40, 157.97 ± 19.55, and 105.23 ± 2.75 mg g^−1^ of total sugars respectively for well‐watered, SD4 stress, and recovered plants; Figure 7A).

In detail, the FDD treatment increased the content of starch in the stems during the drought imposition (FD3, 1.5‐fold more than controls) and after 1 day of rewatering (2.2‐fold more, Figure 7B), while the accumulation of soluble sugars did not change over the experiment (Figure 7C). Plants from SDD treatment accumulated slightly more starch compared with well‐watered conditions (Figure 7B) and significantly more soluble sugars (1.7‐fold more). Interestingly, during the recovery phase, stem sugars in SDD plants return to pre‐stress level within 1 day (Figure 7C).

## 4 DISCUSSION

The two methods adopted to impose drought on potted grapevines were chosen to simulate either (1) a fast water potential decline, rates of ~0.09 (MPa day^−1^), typical of most drought experiments conducted on potted plants (Griesser et al. 2015) or (2) a gradual decline, rates of ~0.025 (MPa day^−1^), more typical of drought under field and natural conditions (Romero et al. 2017). We found significant differences between the fast and slow rates of drought progression among the measured water stress‐related physiological responses, including stomatal conductance, xylem sap ABA concentration, xylem sap pH, and soluble sugar concentrations in xylem sap, as well as modifications in the content of stem carbohydrates. Furthermore, we observed that not only was each drought rate accompanied by distinct xylem biochemical changes, but also the respective changes set the precedent for their corresponding recovery. However, we also observed similarities between the two treatments, particularly in the trajectory of water potential and *g*_*s*_ during the recovery. Taken together, our findings suggest that the evaluation of plant response to stress should be analyzed in the context of plant water potential, while the subsequent response to recovery from stress should be evaluated in the context of time, as xylem pressure immediately returned to pre‐stress levels in both treatments. Moreover, our findings can be analyzed in the context of the impact of drought progression rate on protective strategy (isohydric vs. anisohydric behavior) and the subsequent recovery process.

A binomial method of classification, isohydric and anisohydric (Schultz 2003), has been used to explain differences in stomatal behavior between *Vitis vinifera* varieties in response to water stress. However, this classification should not be decisive (Hochberg et al. 2018), and recently it has been shown that isohydric and anisohydric behavior is not constitutive for a characteristic variety, but it is rather environment‐dependent (Martorell et al. 2015). In general, anisohydric plants withstand a wide variation of water potential but promote photosynthetic gains, thus maintaining higher stomatal aperture and exhibiting a substantial reduction in xylem pressure (Coupel‐Ledru et al. 2017). In contrast, isohydric plants limit water potential variation to protect the hydraulic integrity at the cost of photosynthetic output. They are also more exposed to carbon starvation risk compared with anisohydric plants, due to the prompt stomatal closure in the case of water stress (Tardieu & Simonneau 1998). Grenache typically shows a near‐isohydric response. Indeed, when exposed to FDD, Grenache shut stomata in a step‐like manner in response to small drops in water potential (within 0 to −0.6 MPa). However, Grenache's isohydric behavior disappeared under the SDD treatment. Such dual response contradicts popular approaches that ascribe species or varieties' dominant adaptive stomatal responses to be inherent and independent of environmental conditions (Dal Santo et al. 2016).

Some previous studies have reported switching between isohydric and anisohydric behavior even within the same cultivar (Chaves et al. 2010; Franks et al. 2007; Rogiers et al. 2012; Zhang et al. 2012), but the circumstances promoting this behavioral ambiguity in response to drought remained unclear (Domec & Johnson 2012; Klein 2014). Stress development dynamics may reconcile the apparent inconsistencies and provide insight into the benefits of a shifting strategy adjusting along a stress continuum as opposed to being constrained to an archetypal response. Isohydricity may be a beneficial response under FDD regimes when sudden unanticipated deviations from typical transpirational demand risk exposure to tensions that can endanger xylem functionality (Tyree & Sperry 1988) or accelerate senescence. Under SDD conditions, a near‐anisohydric response can dominate as a slow buildup of tension affords plants the time and security to better acclimate under the reduced risk of sudden hydraulic failure, all the while preserving photosynthetic activity. In this study, Grenache exemplifies such flexibility by modulating stomatal conductance in accordance with drought length and rate. Thus, we suggest that isohydric and anisohydric behaviors bound an array of facultative behaviors imposed by different rates and length of drought stress that permit plants to shift priorities and coordinate responses to optimize the tradeoff between carbon gain and hydraulic function, and analysis of stomatal response to water stress should account for the rate of stress development.

Xylem sap ABA and pH have been shown to mediate stomatal closure and be linked to anisohydric and isohydric behavior (Davies et al. 2002; Marusig & Tombesi 2020; Sharp & Davies 2009). In fact, both strategies are often associated with different degrees of ABA concentration and sensitivity (Coupel‐Ledru et al. 2017). Under FDD, following a moderate decrease in water potential, a steep decline in stomatal conductance was observed along with a large and sudden increase of ABA concentration in xylem sap. Maximum ABA levels were achieved following complete stomatal closure, which may explain the high ABA accumulations in leaves post‐stomatal closure observed in previous studies (Frioni et al. 2020; Tombesi et al. 2015). Under SDD, the increase of ABA concentration was very slow and reached levels 10 times lower than those under the FDD treatment. Despite the stark contrast between the two treatments, in both cases, ABA progressively increased at the onset of drought concurrently to the decline in *g*_*s*_ with complete stomatal closure occurring under similar ABA concentrations (60–80 μg L^−1^). The high accumulation of xylem ABA observed under FDD conditions but not under SDD conditions is a surprising observation suggesting that ABA production by roots in response to stress may be related not to stress severity but to the rate of its occurrence. In addition, the excessive increase in ABA concentration observed in the FDD treatment might suggest lower sensitivity of stomata to ABA concentration under sudden drought (see slight shift in response of *g*_*s*_ to ABA in Figure 5 inset). This might indicate that ABA sensitivity is not an intrinsic varietal property but can be associated with the rate of water stress development. Therefore, ABA sensitivity may not be the best indicator to differentiate between iso/anisohydricity given that stress rate progression might alter ABA sensitivity and patterns of accumulation. We think that this notion is a novel concept that should be further explored as previous studies have found that experimental conditions influence stomatal behavior and apparent sensitivity to ABA (Lavoie‐Lamoureux et al. 2017; Martinez‐Vilalta & Garcia‐Forner 2017).

Moreover, the ABA concentration in the xylem sap and plant sensitivity acquired during stress may also play an important part in stress recovery. There is an increasing appreciation for the fact that the recovery of water potential does not result in immediate stomatal opening (Blackman et al. 2009; Martorell et al. 2014) and a delay is often observed. Such time lag may be an evolved trait that provides additional time for the restoration of hydraulic plant capacity (Martorell et al. 2014; Pagliarani et al. 2019). This delay has been associated with lingering ABA concentrations post‐tension‐release (Brodribb & McAdam 2013; Lovisolo et al. 2008). Indeed, in the present study, we observed the presence of lingering ABA following rehydration. One might expect that gas exchange recovery from fast‐induced stress would be hastened by the quick improvement in water potential. Surprisingly, the recovery of leaf gas exchange was slow and not related to the recovery of water potential in both drying regimes despite levels of lingering ABA being drastically lower in SDD than in FDD. It can be speculated that recovery in Grenache might be linked to its sensitivity to ABA and xylem sap pH acclimation. Photosynthetic recovery is an important competitive advantage of any species; thus, the observed delay can be seen as a disadvantageous behavior. However, this delay might be necessary to assure that xylem transport capacity is restored to its maximum before an increase in transpiration demand. It is imperative to reconsider the way we represent recovery: from assessing it in terms of water potential to looking at it from the perspective of time passing.

**FIGURE 8:**
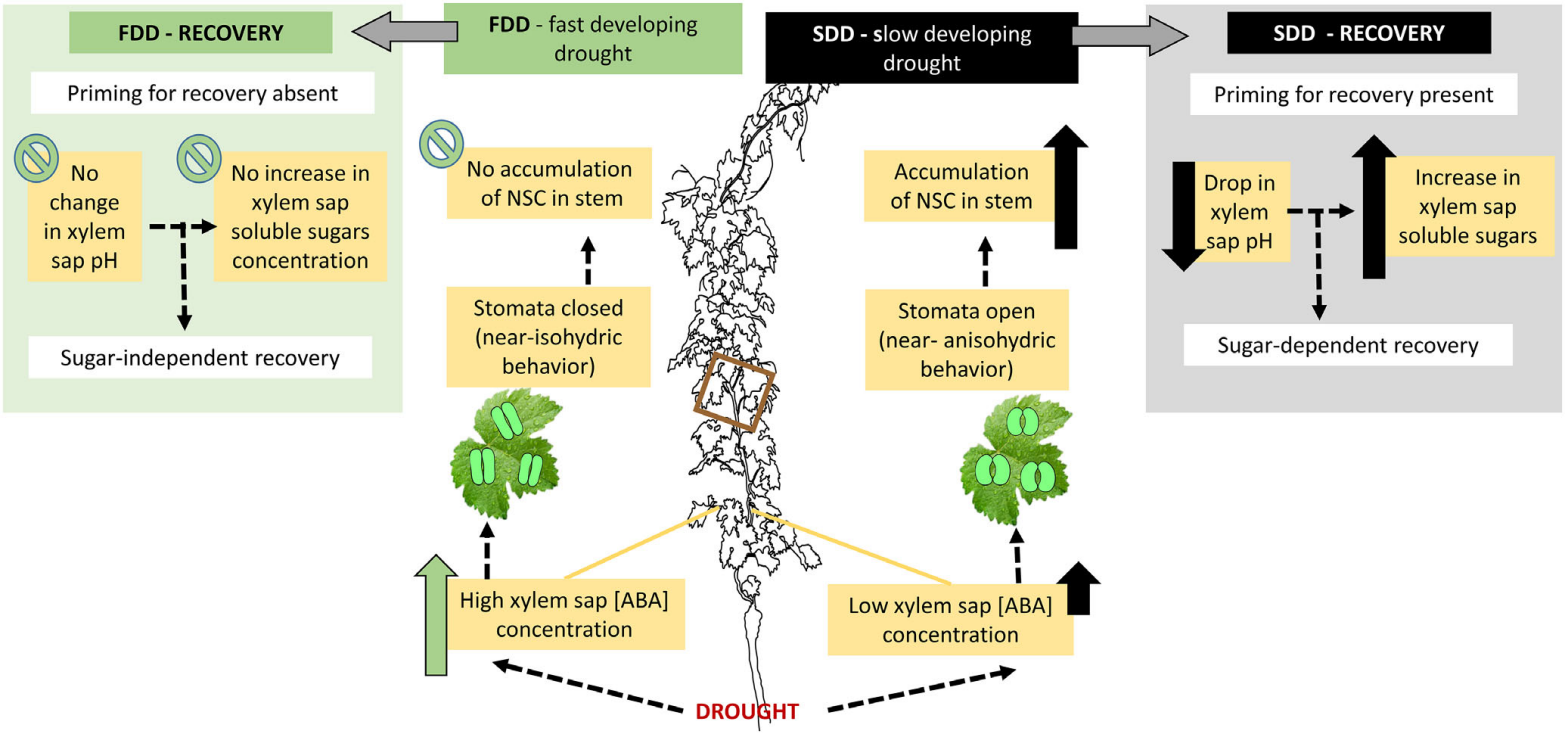
Schematic illustration of grapevine behaviors under fast‐developing drought (FDD; on the left) and slow‐developing drought (SDD; on the right) and under recovery from both stresses. Green (FDD) and black (SDD) arrows indicate increasing or decreasing concentration of the measured parameters. Dotted arrows connect the effects derived from exposing plants to the two different rates of stress and the final consequences on recovery. For the details, please refer to the text

Although in this study we did not assess hydraulic losses due to tension, applied stress was shown to cause embolism in grapevine (Brodersen et al. 2013; Brodersen et al. 2018; Pratt et al. 2020; Tombesi et al. 2014). Furthermore, it has been shown that recovery processes resulting in the restoration of hydraulic capacity require both energy and time to utilize the sudden occurrence of high water potential (Salleo et al. 2004; Secchi & Zwieniecki 2016; Savi et al. 2016; Trifilò et al. 2017). As drought decreases photosynthetic output and growth, it is thought that the NSC storage pool may initially increase due to a reduction in sink activities but subsequently decrease due to the expenditure required to maintain metabolic activity (Trifilo et al. 2017). It might be expected that such behavior would be more pronounced in SDD as a slow decrease in plant water potential would allow more time between the halting of growth and total stomata shutdown, while both growth and stomata shutdown may occur almost simultaneously in FDD and no accumulation should be detected. Indeed, Grenache had increased soluble sugar and starch contents under SDD conditions and did not change sugar content under FDD conditions even if an increase in starch level was observed. It is assumed that the restoration of xylem functional capacity post‐stress exposure requires a pH‐driven accumulation of sugars in xylem sap, which creates an osmotic gradient that stimulates embolism recovery (Salleo et al. 2004; Secchi & Zwieniecki 2016). Such dependency has been previously observed and further supports the notion that, under natural drought conditions, the pH of xylem sap stimulates an efflux of soluble sugars to the xylem (Secchi et al. 2017). Interestingly, during SDD, this accumulation of sugar in sap was imperceptible; however, during recovery, there was a significant increase associated with a drop in pH (Figure 4). Nevertheless, this increase only persisted for a few days. In FDD, no significant changes in xylem sap soluble sugar levels were detected and no relationship between sap pH and SC concentration was present. This differential response between SDD and FDD may suggest that SDD results in physiological preparations aimed at reinstating their hydraulic system, while FDD (most likely not a realistic drought treatment) can result in artifactual responses that may not facilitate full physiological recovery. Taken together, it seems that the length and rate of drought stress affect xylem sap soluble sugar concentration such that longer and slower stress stimulates the processes associated with recovery from embolism, while fast stress progression may hinder the physiological preparations for recovery. See the scheme presented Figure 8 for an overview of the mechanisms developed by Grenache depending on the stress progression rate.

## 5 CONCLUSIONS

- In the case of grenache, isohydric and anisohydric behavior are facultative responses that can be linked to the rate of drought progression. Isohydric behavior protects plants from a sudden increase in tension, while anisohydric response can be linked to a more gradual tension increase that promotes the maintenance of photosynthetic activity.
- Stress progression rate affects the xylem sap ABA concentration and sensitivity of stomata to ABA: high concentrations and lower sensitivity in FDD and low concentrations and high sensitivity in SDD.
- Post‐stress recovery occurs in two phases: (1) fast (hours) recovery of water potential and (2) slow (days) yet continuous recovery of stomatal conductance. The recovery rate was independent of the stress progression rate and could be linked to lingering ABA concentrations in xylem sap and respective sensitivities.
- The concentration of stem NSC was minimally affected by stress progression rates. However, xylem sap soluble sugar content increased in SDD in correspondence to lower pH, suggesting that slow‐developing stress might prime plants for restoring hydraulic capacity.
- The differential response between FDD and SDD (reflected in levels of xylem sap ABA concentration, pH, and NSC) underlines the importance of applying an adequate drying method to better simulate the timing of naturally occurring drought within the studied system.
- During the recovery, the similarities in water potential and *g*_*s*_ suggest that the drying method is less important to the rate of drought recovery, although the nuances of the recovery may be different (stem priming).

## AUTHOR CONTRIBUTION

Cristina Morabito and Jessica Orozco contributed equally and should be considered first authors. Cristina Morabito and Jessica Orozco planned and designed the research with assistance of Francesca Secchi and M. A. Zwieniecki. Cristina Morabito, Jessica Orozco, Giulia Tonel, Silvia Cavalletto and Francesca Secchi performed the physiological and chemical experiments. Giovanna Roberta Meloni conducted the ABA quantification. Cristina Morabito, Jessica Orozco, Andrea Schubert, MAZ and Francesca Secchi contributed to the analyses and discussion of data. Jessica Orozco, Cristina Morabito wrote first version of the manuscript with assistance of Francesca Secchi and M. A. Zwieniecki. All authors contributed to final revision. M. A. Zwieniecki and Francesca Secchi contributed equally.

## Supporting information


**Figure S1** Schematic representation of the experimental setup
**Figure S2** Daily water loss through the imposed droughts
**Figure S3** Net photosynthesis (*A*_*n*_) in relation to stomatal conductance (*g*_*s*_)
**Figure S4** Xylem pressure, abscisic acid (ABA) concentration, pH values, and soluble sugar content measured in the xylem sap of FDD and SDD plants
**Figure S5** Total carbohydrates, starch, and soluble sugars content measured in stem tissues collected from FDD and SDD plants.
**Table S1** Daily average temperature and daily relative humidity

## Data Availability

The data that supports the findings of this study are available in the supplementary material of this article.
