# Supplementary material for: Do the ends justify the means? Impact of drought progression rate on stress response and recovery in Vitis vinifera
Source: Physiol Plant. 2021 Nov 10;174(1):e13590. doi: 10.1111/ppl.13590 (PMC9299143; doi:10.1111/ppl.13590)
Supplement: Supplementary file 1 — Figure S1 Schematic representation of the experimental setup Figure S2 Daily water loss through the imposed droughts Figure S3 Net photosynthesis (A n ) in relation to stomatal conductance (g s ) Figure S4 Xylem pressure, abscisic acid (ABA) concentration, pH values, and soluble sugar content measured in the xylem sap of FDD and SDD plants Figure S5 Total carbohydrates, starch, and soluble sugars content measured in stem tissues collected from FDD and SDD plants. Table S1 Daily average temperature and daily relative humidity [file PPL-174-0-s001.pdf]

Supporting Information

Fig.S1 Schematic representation of the adopted experimental design

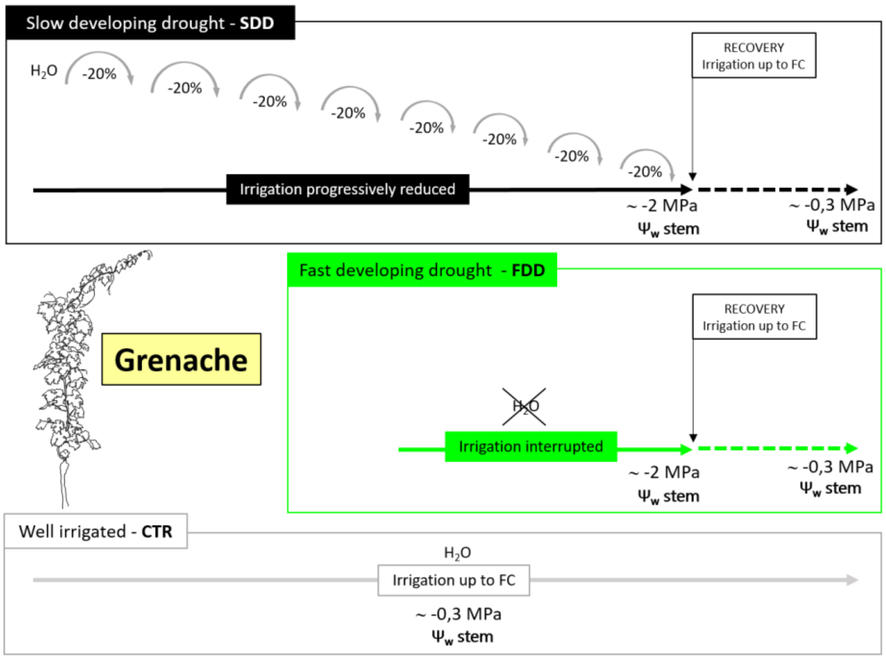

Fig.S2 Daily water loss through the imposed slow developing drought (SDD, black circles) and fast developing drought (FDD; green circles) in Grenache plants.

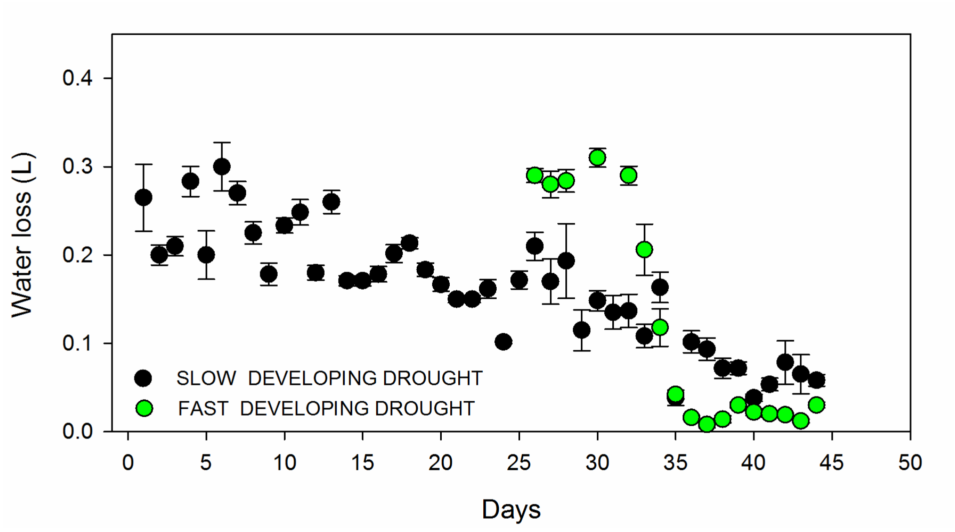

Fig.S3 Net photosynthesis (An) in relation to stomatal conductance (gs) during (a) slow and fast developing drought (SDD: black circles and FDD: green circles) followed by (b) recovery (REC) from slow and fast developing drought treatments. White circles represent well-irrigated plants (CTR).

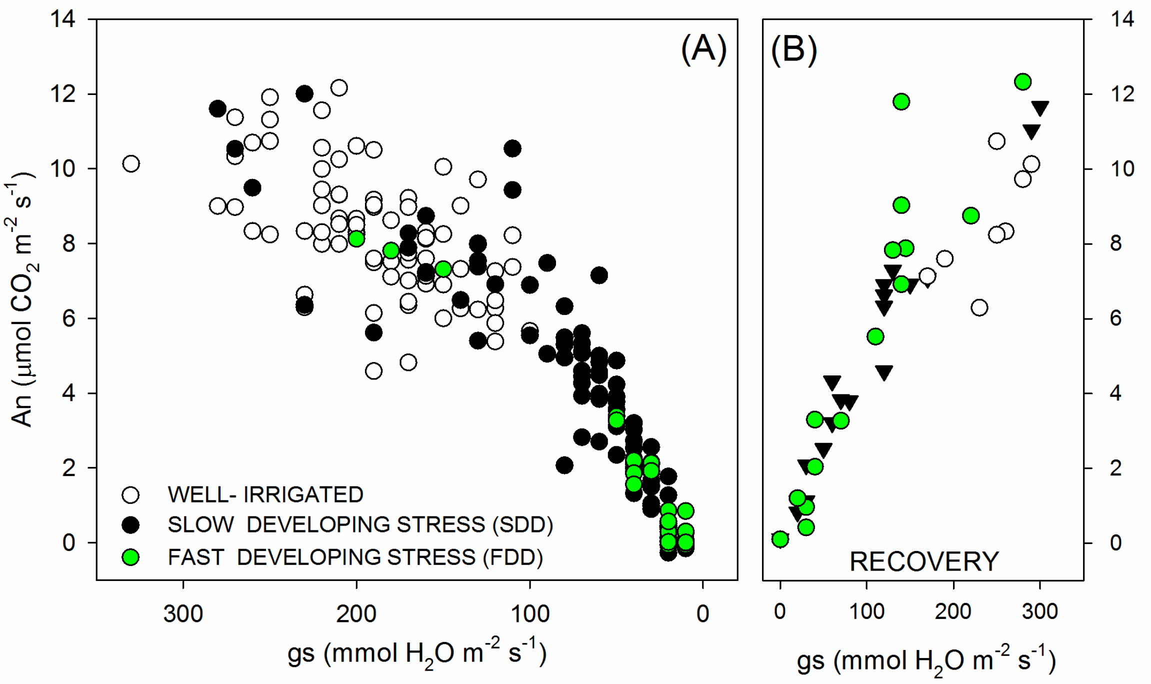

Fig.S4 (a) Xylem pressure, (b) abscisic acid (ABA) concentration, (c) pH values and (d) soluble sugar content measured from xylem sap collected from plants exposed to a fast developing drought (FDD; green bars) and to a slow developing drought (SDD; black bars). One-way ANOVA tests suggest significant differences in both SDD and FDD stressed groups ($P < 0.05$). Letters denote homogeneous groups based on the Fisher LSD method; data are mean values ($n = 3$) and bars are SE.

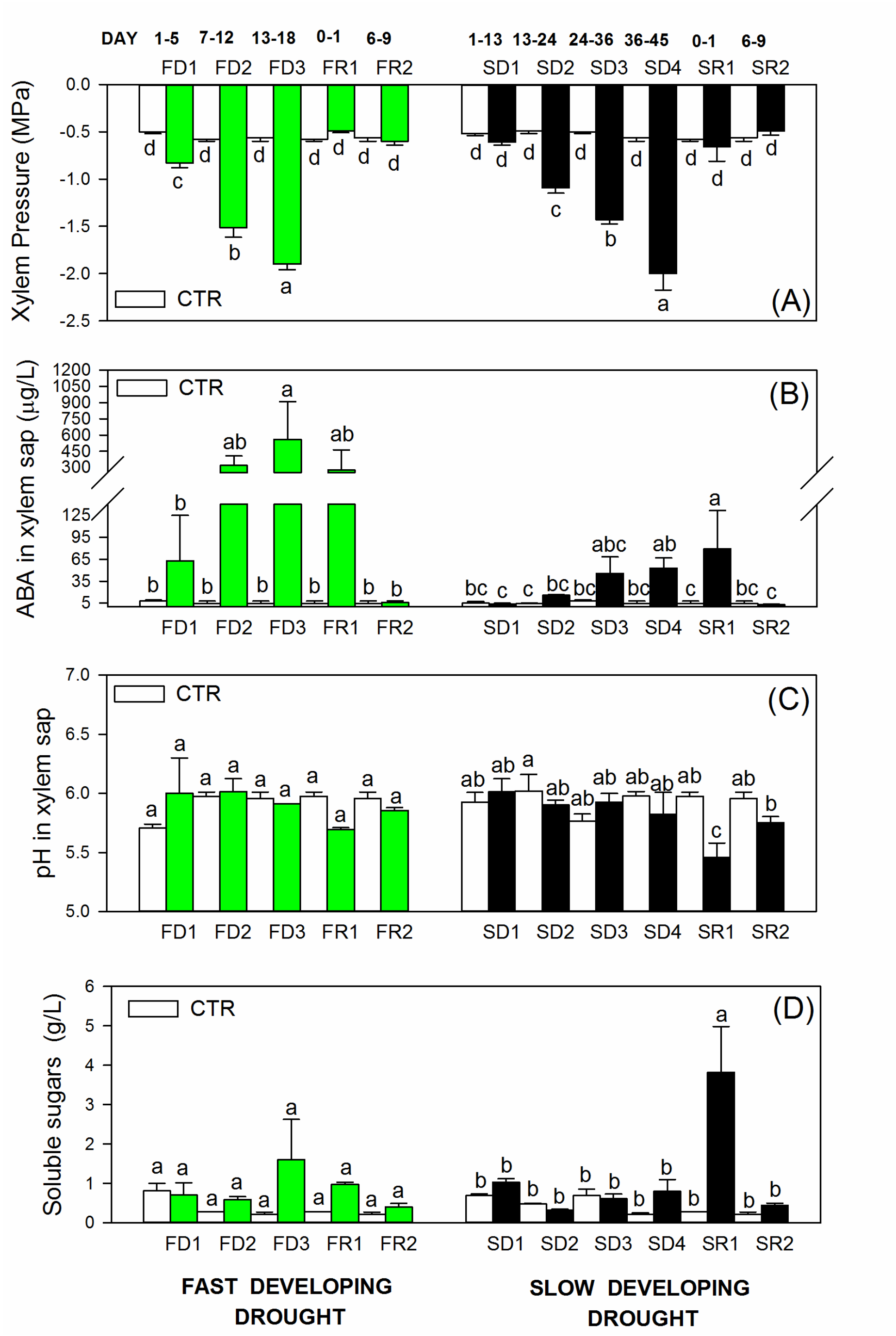

Fig.S5 (a) Total carbohydrates (starch plus soluble sugars), (b) starch content and (c) soluble sugars content measured from stem tissues collected from plants exposed respectively to a fast developing drought (FDD; green bars) and to a slow developing drought (SDD; black bars). One-way ANOVA tests suggest significant differences in both SDD and FDD stressed groups ($P < 0.05$). Letters denote homogeneous groups based on the Fisher LSD method; data are mean values (n = 3) and bars are SE.

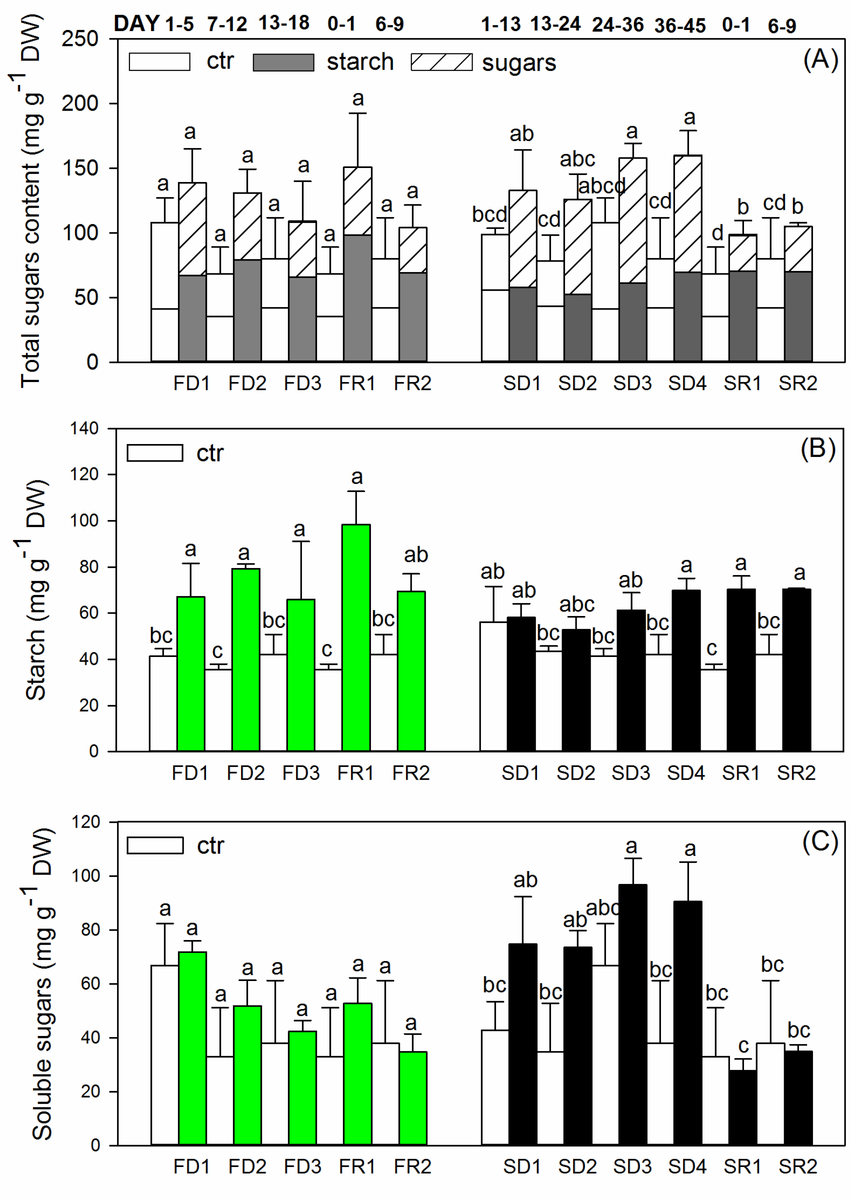

Table S1 Daily average temperature and daily relative humidity recorded in the greenhouse during the experiment.

Treatment	Day.	Daily T (˚C)	sd	T max (˚C)	sd	T min (˚C)	sd	Daily RH (%)	sd	RH max (%)	sd	RH min(%)	sd
stress	1	26,08	5,82	26,94	6,13	25,28	5,30	48,46	12,85	51,41	11,24	45,66	13,50
stress	2	26,53	6,50	27,29	6,83	25,69	6,21	53,01	17,97	56,33	17,07	50,43	18,52
stress	3	26,99	6,00	27,82	6,29	26,19	5,55	41,12	10,15	43,74	9,32	39,04	10,73
stress	4	28,08	6,80	29,09	7,12	27,15	6,35	44,27	14,06	46,97	13,17	41,12	14,24
stress	5	29,01	7,65	30,00	8,02	28,02	7,25	48,51	16,77	51,61	15,77	45,35	17,01
stress	6	28,56	5,86	29,42	6,27	27,78	5,51	57,66	11,95	60,81	12,06	54,97	12,26
stress	7	27,73	4,32	28,58	4,81	27,08	3,88	56,28	8,83	58,98	7,52	52,84	9,17
stress	8	27,17	6,49	28,13	6,81	26,31	6,13	67,79	20,32	71,43	19,08	63,54	21,46
stress	9	27,73	6,70	28,76	6,93	26,88	6,36	64,53	19,61	68,33	18,24	60,39	20,68
stress	10	25,28	4,66	26,37	5,28	24,27	3,97	74,52	18,72	79,24	16,19	70,01	20,36
stress	11	27,50	6,90	28,38	7,24	26,64	6,58	63,01	24,41	66,80	23,05	59,80	25,89
stress	12	28,79	6,68	29,80	7,21	27,81	6,17	48,11	14,84	52,55	14,98	44,53	14,69
stress	13	28,57	6,43	29,56	6,78	27,67	5,97	51,64	14,80	54,67	13,33	48,69	14,74
stress	14	27,64	5,17	28,46	5,59	26,91	4,76	58,93	12,90	61,52	11,69	56,03	13,62
stress	15	28,11	5,08	28,92	5,44	27,35	4,60	62,74	14,89	65,77	13,34	59,46	15,77
stress	16	25,99	4,25	26,87	4,76	25,34	3,87	66,81	14,80	69,72	13,51	63,80	15,71
stress	17	24,90	4,19	25,73	4,63	24,27	3,69	75,33	17,43	78,64	14,71	71,81	18,13
stress	18	26,67	6,28	27,52	6,80	25,79	5,80	70,57	22,73	76,65	20,49	67,89	24,34
stress	19	26,01	4,69	26,88	5,27	25,24	4,05	72,55	13,70	75,42	11,61	68,45	15,11
stress	20	26,54	6,24	27,46	6,69	25,57	5,55	72,35	19,95	76,02	18,21	68,81	21,20
stress	21	22,58	2,77	23,10	2,82	22,17	2,54	78,67	13,90	81,67	11,83	74,98	14,37
stress	22	26,14	7,76	27,07	8,28	25,19	7,37	66,49	28,78	70,50	27,42	63,26	31,03
stress	23	28,11	7,28	29,13	7,79	27,20	6,91	55,45	21,22	58,93	20,03	52,43	21,60
stress	24	28,92	6,63	29,86	7,00	28,05	6,28	55,93	15,62	59,02	14,44	52,35	16,45
stress	25	27,64	5,53	28,75	5,89	26,83	5,01	64,14	14,46	67,92	12,09	60,17	14,21
stress	26	23,88	4,63	24,77	5,43	23,15	3,94	77,99	17,49	81,23	15,76	74,02	19,33
stress	27	26,07	6,57	26,93	7,00	25,23	6,17	65,31	23,71	69,43	22,09	62,78	25,42
stress	28	27,56	6,87	28,50	7,30	26,70	6,55	54,17	19,44	57,51	19,02	51,26	19,78
stress	29	28,30	5,67	29,19	6,22	27,45	5,11	56,04	12,17	58,57	11,77	52,58	12,45
stress	30	29,58	6,44	30,50	6,80	28,77	6,06	51,43	15,68	54,16	15,53	48,94	15,80
stress	31	28,02	7,44	29,09	7,83	27,11	6,92	57,65	22,76	60,10	22,22	53,58	22,06
stress	32	27,78	5,92	28,49	6,32	26,98	5,54	69,18	21,48	72,51	20,85	66,53	22,33
stress	33	25,74	3,84	26,62	4,24	25,00	3,18	78,86	15,42	82,03	13,13	75,35	16,20
stress	34	27,05	5,76	27,74	6,19	26,21	5,26	74,60	21,25	77,87	19,85	72,24	22,80
stress	35	29,44	6,74	30,34	7,14	28,54	6,34	63,46	18,32	66,22	17,59	60,61	18,61
stress	36	30,13	6,25	30,97	6,64	29,23	5,82	59,01	18,84	62,57	17,76	56,54	19,84
stress	37	28,87	5,01	29,72	5,39	28,13	4,42	59,42	11,02	62,59	10,04	56,39	11,63
stress	38	30,00	7,27	30,96	7,68	28,98	6,77	52,72	15,27	55,35	14,42	49,40	15,18
stress	39	29,27	5,19	30,31	5,87	28,37	4,54	64,11	14,36	66,60	13,13	61,21	15,54
stress	40	30,36	5,91	31,23	6,33	29,46	5,37	61,98	19,00	65,66	18,68	59,15	19,12
stress	41	30,45	5,86	31,29	6,24	29,65	5,37	54,85	14,04	57,65	13,84	52,62	14,23
stress	42	29,66	6,04	30,57	6,46	28,74	5,41	50,93	10,12	53,99	9,30	48,64	10,45
stress	43	28,44	5,94	29,39	6,24	27,62	5,47	60,27	16,39	63,17	16,30	56,60	16,17
stress	44	27,41	5,97	28,35	6,56	26,59	5,50	61,47	17,79	64,42	16,90	58,42	18,34
recovery	1	25,77	4,81	26,48	5,17	25,08	4,28	64,62	14,84	67,58	13,61	61,41	14,97
recovery	2	27,65	6,59	28,48	7,03	26,72	6,08	60,80	19,44	63,72	18,61	58,51	20,62
recovery	3	28,26	6,05	29,26	6,63	27,36	5,52	56,67	17,38	59,49	16,46	54,06	18,07
recovery	4	27,43	4,94	28,11	5,25	26,69	4,46	63,04	12,44	65,17	11,37	60,60	12,80
recovery	5	25,02	3,01	25,63	3,21	24,46	2,41	75,27	12,21	77,80	10,16	72,13	12,86
recovery	6	26,11	5,66	26,89	6,15	25,27	5,14	68,95	20,81	71,93	20,03	66,70	22,48
recovery	7	26,84	5,38	27,68	5,92	25,95	4,58	62,02	16,03	65,01	14,68	59,22	16,75
recovery	8	28,00	6,20	28,71	6,47	27,04	5,52	57,43	18,72	60,15	17,91	55,25	19,04
recovery	9	27,22	4,85	28,15	5,32	26,45	4,23	59,76	13,58	62,82	13,28	55,86	13,15
recovery	10	27,84	6,27	28,56	6,56	26,77	5,42	60,55	18,05	63,88	17,03	57,83	18,40
